# Supplementary material for: Integrated Expression Analysis of Small RNA, Degradome and Microarray Reveals Complex Regulatory Action of miRNA during Prolonged Shade in Swarnaprabha Rice
Source: Biology (Basel). 2022 May 23;11(5):798. doi: 10.3390/biology11050798 (PMC9138629; doi:10.3390/biology11050798)
Supplement: Supplementary file 1 [file biology-11-00798-s001.zip › Legends-Supplemenatry Figures.pdf]

Supplementary Table S1–S18. Assorted Data of Details of candidates of miRNA sequencing, Degradome sequencing and Microarray done with Panicle samples of Swarnaprabha rice after 20 days of shade.

Supplementary Figure S1. Prediction of secondary structure of five novel miRNA expressed under prolonged shade. Sequences coloured yellow represent the mature novel miRNA generated from 5′ and 3′ arm miRNA precursors. The nucleotides are numbered on the precursor. (A) mireap-m0004, (B) mireap-m0001-3p, (C) mireap-m0014-5p, (D) mireap-m0007-5p and (E) mireap-m0005-5p. 5′ loaded adenine or uridine is coloured in Pink. The sequences of these miRNA are present in Supplementary Table 4 in **Bold** Letters.

Supplementary Figure S2. Family Distribution of known miRNA from Swarnaprabha panicle samples. Total 411 known miRNA were grouped into 64 different families as described in Suppl Table 6. Top 10 MIR families are represented.

Supplementary Figure S3. Differential expression analysis of the novel miR (DEm) from sun and shade grown samples. Volcano plot of (A) the novel DEm. Heat-map analysis of (B) the novel DEm. Colour codes represent the differential regulation of the miRNA. Differential expression was based on Log_2 Fold-change ($p \leq 0.9$).

Supplementary Figure S4. Pictorial representation of method used for data analysis. The figure represents the analysis work flow for selection of candidate miRNA and target genes. It represents an integrative analysis in SP including miRNA sequencing, whose targets were validated using degradome sequencing, further filtered from microarray expression data and selected candidates were verified using qRT-PCR. The Red arrows indicate the sequential work flow. The criteria of selection are in smaller font, Boxed items are steps of analysis, Circled items are connections to the next procedure, numbers within brackets are no. of candidate miRNA or target transcripts.

Supplementary Figure S5. Pathway enrichment analysis of predicted targets of the DEm. Upper and lower row represents biological process (BP), cellular component (CC) and molecular function (MF) from sun and shade libraries respectively. Colour code represents significance, X-axis represents gene ratio.
