# Supplementary figures and images for: Integrated Expression Analysis of Small RNA, Degradome and Microarray Reveals Complex Regulatory Action of miRNA during Prolonged Shade in Swarnaprabha Rice
Source: Biology (Basel). 2022 May 23;11(5):798. doi: 10.3390/biology11050798 (PMC9138629; doi:10.3390/biology11050798)

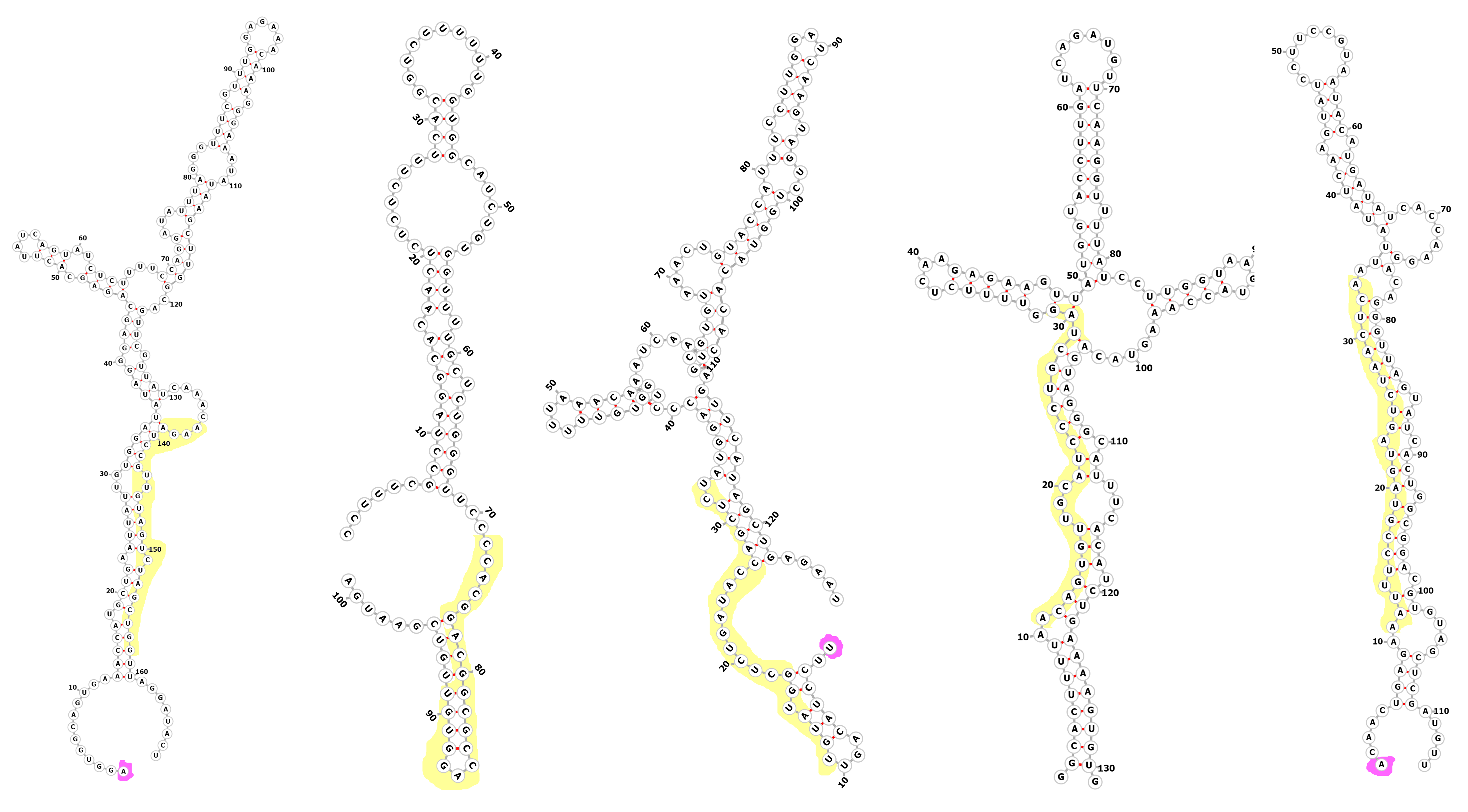

Supplement: Supplementary file 1 [file biology-11-00798-s001.zip › Figure S1.tif]

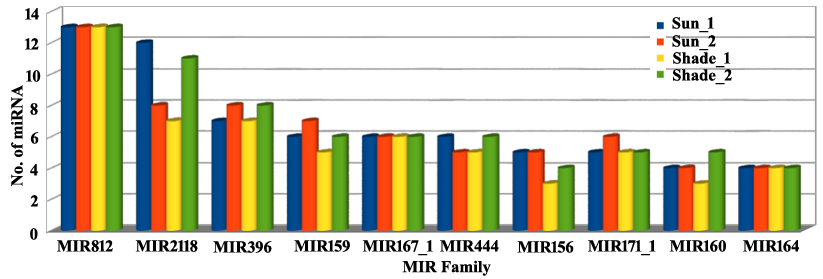

Supplement: Supplementary file 1 [file biology-11-00798-s001.zip › Figure S2.tif]

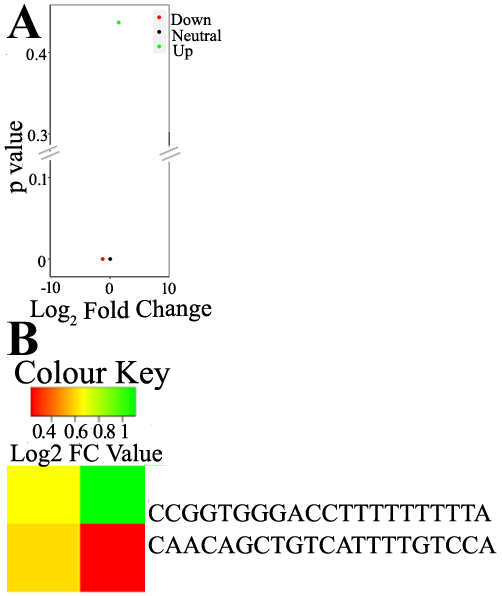

Supplement: Supplementary file 1 [file biology-11-00798-s001.zip › Figure S3.tif]

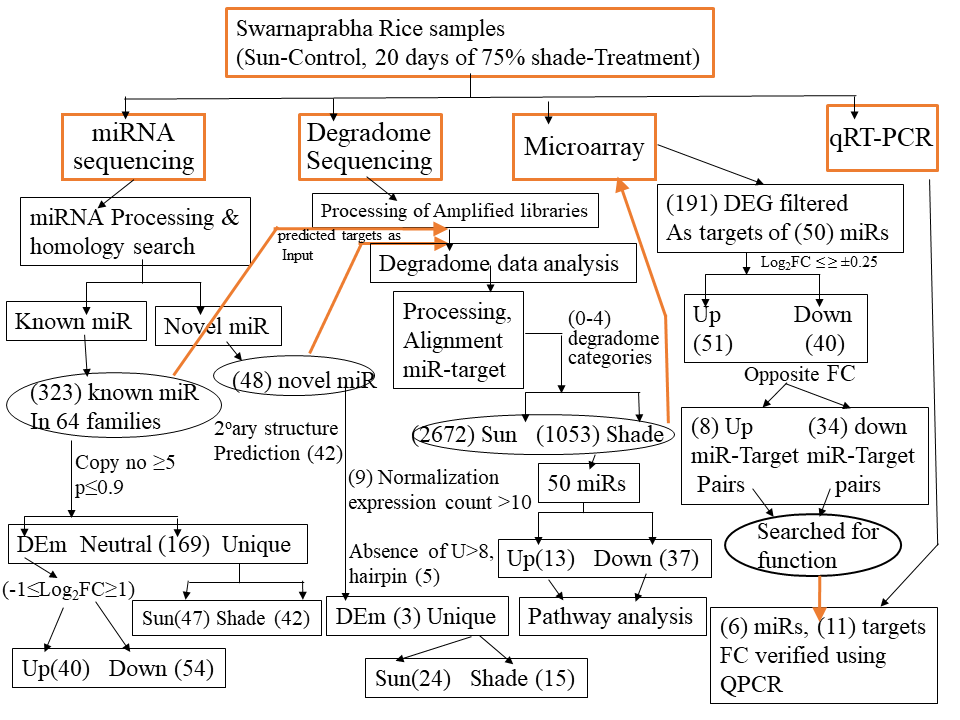

Supplement: Supplementary file 1 [file biology-11-00798-s001.zip › Figure S4.tif]

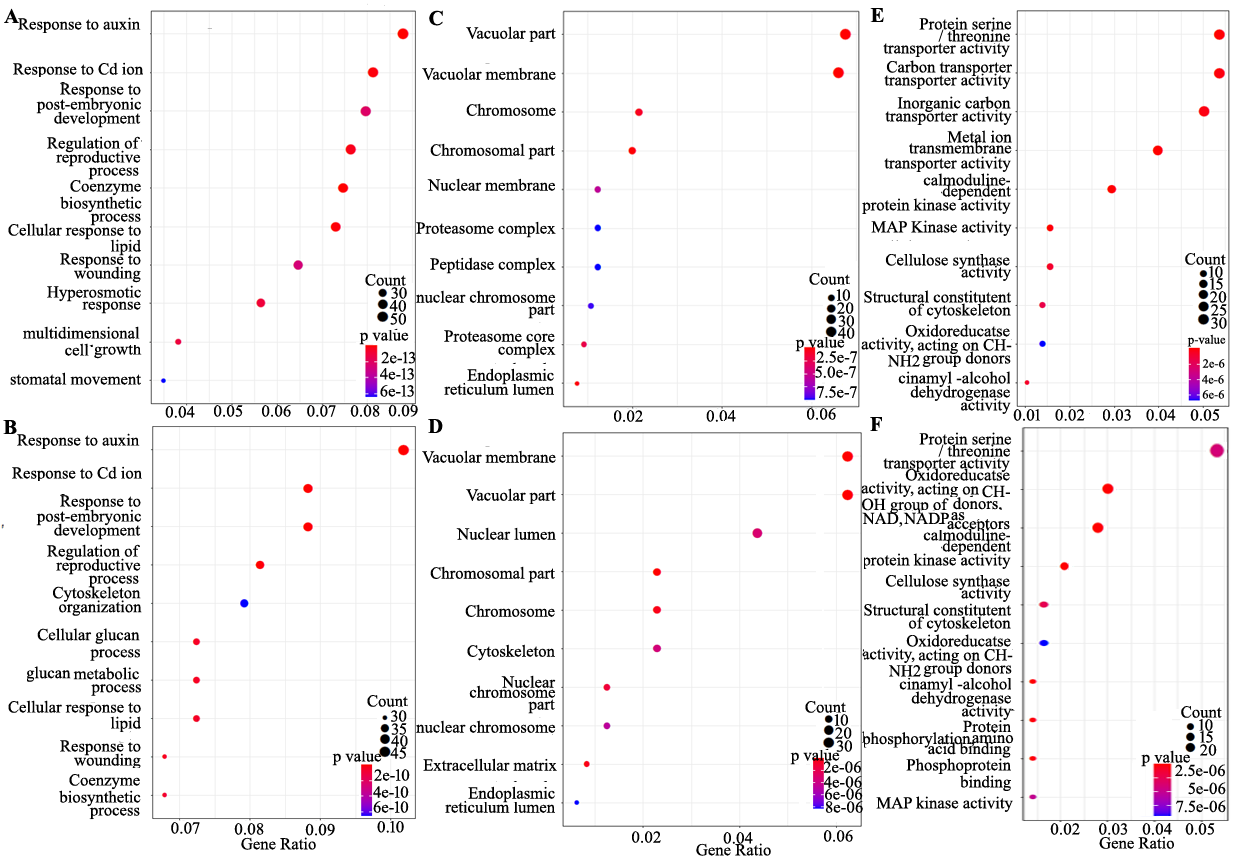

Supplement: Supplementary file 1 [file biology-11-00798-s001.zip › Figure S5.tif]
